# Identification of genomic regions associated with inbreeding depression in Holstein and Jersey dairy cattle

**DOI:** 10.1186/s12711-014-0071-7

**Published:** 2014-11-18

**Authors:** Jennie E Pryce, Mekonnen Haile-Mariam, Michael E Goddard, Ben J Hayes

**Affiliations:** Biosciences Research Division, Department of Environment and Primary Industries Victoria, 5 Ring Road, Bundoora, 3083 Australia; Dairy Futures Cooperative Research Centre, 5 Ring Road, Bundoora, VIC 3083 Australia; La Trobe University, Bundoora, VIC 3086 Australia; Department of Agriculture and Food Systems, University of Melbourne, Parkville, 3010 Australia

## Abstract

**Background:**

Inbreeding reduces the fitness of individuals by increasing the frequency of homozygous deleterious recessive alleles. Some insight into the genetic architecture of fitness, and other complex traits, can be gained by using single nucleotide polymorphism (SNP) data to identify regions of the genome which lead to reduction in performance when identical by descent (IBD). Here, we compared the effect of genome-wide and location-specific homozygosity on fertility and milk production traits in dairy cattle.

**Methods:**

Genotype data from more than 43 000 SNPs were available for 8853 Holstein and 4138 Jersey dairy cows that were part of a much larger dataset that had pedigree records (338 696 Holstein and 64 049 Jersey animals). Measures of inbreeding were based on: (1) pedigree data; (2) genotypes to determine the realised proportion of the genome that is IBD; (3) the proportion of the total genome that is homozygous and (4) runs of homozygosity (ROH) which are stretches of the genome that are homozygous.

**Results:**

A 1% increase in inbreeding based either on pedigree or genomic data was associated with a decrease in milk, fat and protein yields of around 0.4 to 0.6% of the phenotypic mean, and an increase in calving interval (i.e. a deterioration in fertility) of 0.02 to 0.05% of the phenotypic mean. A genome-wide association study using ROH of more than 50 SNPs revealed genomic regions that resulted in depression of up to 12.5 d and 260 L for calving interval and milk yield, respectively, when completely homozygous.

**Conclusions:**

Genomic measures can be used instead of pedigree-based inbreeding to estimate inbreeding depression. Both the diagonal elements of the genomic relationship matrix and the proportion of homozygous SNPs can be used to measure inbreeding. Longer ROH (>3 Mb) were found to be associated with a reduction in milk yield and captured recent inbreeding independently and in addition to overall homozygosity. Inbreeding depression can be reduced by minimizing overall inbreeding but maybe also by avoiding the production of offspring that are homozygous for deleterious alleles at specific genomic regions that are associated with inbreeding depression.

**Electronic supplementary material:**

The online version of this article (doi:10.1186/s12711-014-0071-7) contains supplementary material, which is available to authorized users.

## Background

Inbreeding depression is the reduction in fitness of offspring that result from the mating between individuals that share at least one common ancestor. Inbreeding reduces fitness by increasing the number of homozygous deleterious recessive alleles that affect traits related to fitness, such as survival, disease resistance, predation and birth weight in avian and mammalian populations [1]. Some published examples include a dramatic effect on offspring survival and reproduction in both mice [2] and freshwater snails [3]. In livestock and poultry species, preventing inbreeding is important because it is associated with a reduction in animal performance and consequently profitability [4-7] and with an increased frequency of genetic defects [8]. Ideally, livestock breeding programs increase the frequency of favourable alleles and possibly their fixation, while minimizing homozygosity for deleterious recessive alleles. With the availability of genotyping data based on high-density SNP (single nucleotide polymorphism) chips, these objectives become possible.

Genetic markers can be used to estimate inbreeding as the realised proportion of the genome that is identical by descent (IBD), for example by calculating a genomic relationship matrix (GRM) among individuals [9-11]. The expected value of a diagonal element of the GRM, conditional on the pedigree of the animal, is 1 + F where F is the inbreeding coefficient of the animal. Unlike pedigree-based inbreeding, a genomic estimate of inbreeding does not suffer from lack of depth of pedigree data and pedigree errors and it measures realised inbreeding that can vary between animals that have the same pedigree, i.e. full sibs.

The diagonal elements of the GRM and the proportion of the genome where SNPs are homozygous are both examples of genomic measures that can be used to estimate the level of inbreeding across the genome. A limitation of both of these measures is that they do not distinguish between identity by state and identity by descent. One possibility to resolve this is to use runs of homozygosity (ROH), which are regions of the genome where the copies inherited from the two parents are IBD [12]. ROH can be used to investigate genome-wide inbreeding and to identify the localization of specific regions of the genome that are IBD. Compared to the diagonal or the GRM or proportion of homozygous SNPs, long ROH are unlikely to have arisen by chance, and are more likely to be stretches of homologous chromosomes within the same individual that are IBD [13]. In fact, the length of ROH inversely correlates to the distance in the pedigree where a common ancestor is present [14], which makes it a powerful method to detect inbreeding effects [15]. ROH are shorter when the common ancestor that gave rise to the inbreeding arose many generations back (distant inbreeding), while they are longer when inbreeding occurred more recently [16]. Inbreeding arising from a distant common ancestor should have less effect on fitness compared with inbreeding from a recent common relative because natural selection over long periods of time should act to purge deleterious alleles from the population [17]. For example, in mice, the effect of inbreeding depression on litter size has been observed to be greater for inbreeding from recent compared with more distant common ancestors [18].

Dairy cattle are potentially a good model to investigate the effects of genome-wide and genome location-specific inbreeding depression, since phenotypes of traits associated with fitness (e.g., fertility) and quantitative performance traits (such as milk yield) are measured on a large scale. In addition, intense selection of males to be the sires of the next generation has made it almost impossible to find dairy cattle without multiple common ancestors [19], so there is substantial variation in the level of inbreeding among individuals in dairy populations.

In this study, we used data on Holstein and Jersey dairy cows with pedigree data and phenotypes for milk production and fertility traits and for which a subset had SNP genotypes to:

1. Estimate inbreeding depression for milk production and fitness traits using pedigree-based inbreeding coefficients.
2. Compare these estimates to inbreeding depression estimated from genome-wide estimates that were derived from SNP data.
3. Investigate whether there are specific regions of the genome that are associated with inbreeding depression in fertility and milk production.

## Methods

### Pedigree-based inbreeding coefficients

Pedigree data that traced back to the 1950s were available on 2 070 219 Holstein and 293 588 Jersey cows [20]. Animals with unknown parents were assigned to genetic groups based on their birth year, country of origin and sex, following standard Australian Dairy Herd Improvement Scheme (ADHIS; Melbourne, Australia) genetic evaluation procedures [20].

Only cows with at least two generations of complete pedigree and born after 1994 were retained. The proportion of cows with complete pedigree over the first four generations was approximately 0.75 in both the Holstein and Jersey populations and was derived using the following equation [21,22]:

$$ C=\frac{1}{d}{\displaystyle \sum_{i=1}^d{a}_i} $$

where *C* is the pedigree completeness, *d* is the number of generations and *a*_*i*_ is the proportion of known ancestors in generation *i*. Selection of cows was restricted to animals born in or after 1994 to be able to compare the dataset with only pedigree information with the genotyped population. After editing, pedigree-based inbreeding coefficients were calculated using the algorithm of Meuwissen and Luo [23] for 338 696 Holstein and 64 049 Jersey individuals.

### Genomic inbreeding coefficients

The genotyped cows that were included in this study were part of an initiative of the Dairy Future’s Cooperative Research Centre (Melbourne, Australia). Briefly, cows were selected to become part of the Australian genomic reference population on the basis of completeness of their phenotypic data. They were also selected to have sires that were genotyped and part of the national Australian genomic reference population. For each cow in the dataset, the validity of its sire was verified by checking for excessive “opposing homozygotes”, which is when an individual’s alleles at a given locus are homozygous, but for different alleles than its sire [24]. The pedigree was corrected in cases for which a parent could be identified [24]. Offspring that had more than 20 genotypes that were incompatible with those of their parents were removed. After quality control, genotypes were available for 8853 Holstein and 4138 Jersey females, which were genotyped with the Illumina BovineSNP50 BeadChip (Illumina, San Diego, CA; [25]) using a previously described quality control method for SNP editing [26]. After editing, 45 753 and 43 737 SNPs remained for the Holstein and Jersey datasets, respectively. The SNPs were ordered by chromosome position using *Bos taurus* build UMD 3.1 (Center for Bioinformatics and Computational Biology, University of Maryland, MD). The average spacing between SNPs retained for this study was 58 kb. Homozygous SNPs were coded as 2 or 0 and heterozygous SNPs as 1.

The following measures of inbreeding were calculated using the genomic data:

1. The genomic relationship of an individual with itself relative to a base population was calculated as the diagonal of the genomic relationship matrix minus 1 (GRM_F) using [9,11]:
  $$ \mathrm{G}\mathrm{R}\mathrm{M}\_\mathrm{F}=\frac{1}{N}{\displaystyle {\sum}_m\frac{{x^2}_m-\left(1+2{p}_m\right){x}_m+2{p^2}_m}{2{p}_m\left(1-{p}_m\right)}}-1, $$
  where *N* is the number of SNPs, *p*_*m*_ is the allele frequency of SNP *m*, and *x*_*m i*_s the genotype code at SNP *m* (0, 1 or 2). Allele frequencies *p*_*m*_ were calculated separately for the Jersey and Holstein populations, which included genotyped males of the same breed, which amounted to a total of 12 649 and 5240 Holstein and Jersey individuals, respectively. Thus, GRM_F was relative to the current population, as represented by the sample of genotyped females and bulls that are part of the Australian genomic reference population.
2. The proportion of the genome that was homozygous (i.e. the proportion of total SNP genotypes that were either AA or BB).
3. The proportion of the genome that consists of runs of homozygosity (ROH_F) that were at least *n* SNPs in length, where *n* ranged from 5 to 100. If the ROH at SNP position *i* exceeded *n*, then *ROH*_*i*_ was coded as 1 or else 0. ROH_F was the sum of *ROH*_*i*_ divided by the total number of SNPs (*nSNP*): $ROH\_F=\frac{{\displaystyle \sum }RO{H}_i}{nSNP}.$

### Phenotypic data

For the Holstein breed, 887 561 lactation records for milk, fat and protein yields were available for 338 696 cows (i.e. each cow had on average 2.6 lactation records) and 755 618 calving interval records (a measure of fertility that records the interval between two consecutive calving dates in days) were available for 299 590 cows. In Australia, calving interval is available on more cows than measures of fertility calculated from insemination and pregnancy diagnosis data [20], which is why calving interval was chosen as the fertility trait for analysis. For the Jersey breed, 179 108 milk, fat and protein yield records (on average 2.8 lactation records per cow) were available for 64 049 animals and 153 347 calving interval records were available for 57 049 animals. Phenotypic means of the traits included in the analysis are in Table 1. The average number of lactation records for cows with genotypes was 3.84 and 3.35 for the Holstein and Jersey data, respectively.

**Table 1:** **Means and standard deviations (SD) of milk, fat and protein yields and calving interval**

	**Holstein**		**Jersey**	
	**Mean**	**SD**	**Mean**	**SD**
Milk (L)	7 286	2 522	5 197	1 635
Fat (kg)	282	99	256	83
Protein (kg)	239	86	195	63
Calving interval (d)	406	69	392	59

### Statistical analyses

All analyses were conducted separately for the Holstein and Jersey datasets. Following previous research using a very similar dataset by Haile-Mariam et al. [20], we used the following model to estimate the effect of inbreeding on the phenotype being analysed:

$$ {y}_{ijklmn}=\mu +HY{S}_i+ parit{y}_j+ mont{h}_k+{\mathrm{b}}_1 ag{e}_{ijklmn}+{\mathrm{b}}_2{F}_{ijklmn}+ per{m}_l+co{w}_m+{e}_{ijklmn} $$

where *y*_*ijklmn*_ is the *n*^*th*^ record of the phenotype (milk, fat or protein yield or calving interval), *HYS*_*i*_ the *i*^*th*^ herd-year-season of calving, *parity*_*j*_ the *j*^*th*^ parity, *month*_*k*_ the *k*^*th*^ month of calving, and b_1_ the regression coefficient on age_ijklmn_ , which was the age at first calving, b_2_ is the regression coefficient on *F*_*ijklmn*_, which was the measure of inbreeding (pedigree, GRM_F, homozygosity, or ROH_F), *perm*_*l*_ is the *l*^*th*^ random permanent environmental effect to account for multiple records on each individual, and *cow*_*m*_ was the random genetic effect for the *m*^*th*^ animal, assumed to follow the distribution N(0, **A**σ^2^), where **A** is the numerator relationship matrix, and *e*_*ijklmn*_ is the random error associated with the observation. The quadratic effects of inbreeding were also tested in preliminary analyses but are not presented since none were significant. ASReml version 3.0 was used for the analysis [27]. Finally, the genomic measures of inbreeding were tested after correcting for pedigree inbreeding by simultaneously fitting pedigree inbreeding and each genomic measure of inbreeding in turn.

The genome-wide association study (GWAS) was designed to investigate the effect of overlapping ROH at consecutive positions across the genome using sliding windows, so the number of analyses was equal to the number of SNPs. Since the effect of inbreeding estimated using ROH, was of interest in this analysis, the model also included the SNP present at the start of the ROH to correct for its additive effect. In this analysis, milk yield was used to represent milk production traits, and calving interval to represent fertility traits. The model used for the GWAS was:

$$ {y}_{ijklmn}=\mu +HY{S}_i+ parit{y}_j+ mont{h}_k+{\mathrm{b}}_1 ag{e}_{ijklmn}+{\mathrm{b}}_3SN{P}_{ijklmn}+{\mathrm{b}}_4RO{H}_{ijklmn}+ per{m}_l+co{w}_m+{e}_{ijklmn} $$

This model was similar to that defined above, except that *F* was replaced by two new covariates: (1) the regression coefficient (b_3_) on *SNP*_*ijklmn*_, which was to correct for the additive effect of the presence of additional copies of the allele, and (2) the regression coefficient (b_4_) on *ROH*_*ijklmn*_, where, at each SNP position, *ROH*_*ijklmn*_ was coded as 1 when a run of homozygosity of at least 50 SNPs was present at *SNP*_*ijklmn*_ for that animal, and as 0 otherwise. A ROH was selected as having a statistically significant association with the trait analyzed if the regression coefficient had a p-value less than 0.001.

To identify possible candidate genes, QTL regions were defined based on SNP positions with significant associations (P <0.001) of ROH with milk yield or fertility. A list of annotated genes between the positions of the first and last SNP in the ROH or cluster of ROH was obtained from Ensembl BioMart MartView (http://asia.ensembl.org/biomart/martview/). QTL regions were also compared to QTL previously identified for the analysed trait through the Cattle QTLdb [28] (http://www.animalgenome.org/cgi-bin/QTLdb/BT/index).

## Results

### Inbreeding coefficients

**Figure 1:**
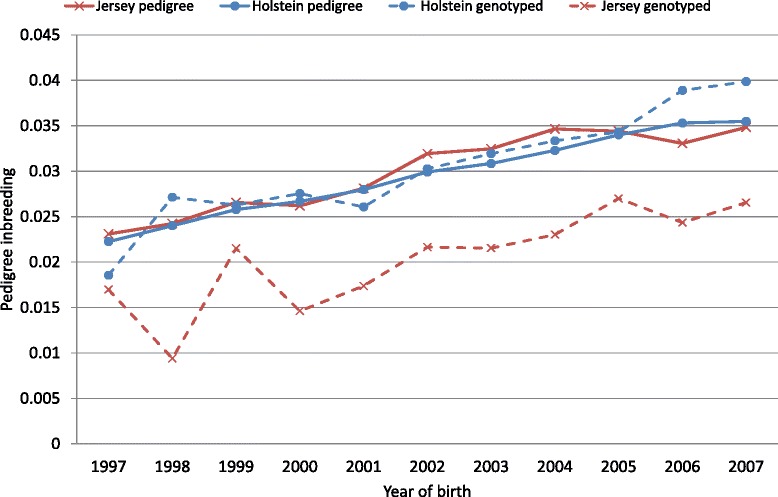
**Pedigree inbreeding coefficients by year of birth for cows born between 1997 and 2007.** Inbreeding coefficients for all animals in the dataset are shown with a solid line; the genotyped subset are shown with a dashed line.

**Figure 2:**
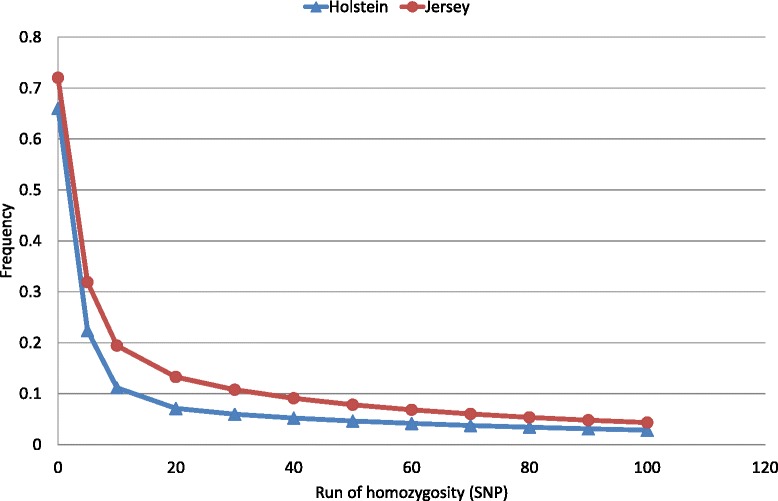
**The average proportion of the genome comprising of runs of homozygosity.**

For the Holstein breed, the mean inbreeding coefficients, calculated using pedigree, were greater for genotyped cows than for the population that had only pedigree-based inbreeding coefficients. However for the Jersey breed, the opposite was true: inbreeding coefficients of genotyped cows were smaller than those of the pedigree recorded population (Table 2; Figure 1). Genomic measures of inbreeding were all higher for the Jersey than for the Holstein population (Table 2). For example, the average proportion of the genome that was homozygous was 0.66 and 0.72 for the Holstein and Jersey populations, respectively. Jersey animals also had longer ROH than Holstein animals (Figure 2). The rate of inbreeding, calculated as the regression of pedigree inbreeding on birth year (1997 to 2007), was 0.13%/year in Holstein cows with pedigree information and 0.18%/year in the genotyped Holstein population. The equivalent rates for the Jersey animals were both 0.13% (Figure 1).

**Table 2:** **Means and standard deviations in parentheses of measures of inbreeding in Holstein and Jersey populations**

	**Holstein**		**Jersey**	
	**All** ^**1**^	**Genotyped** ^**2**^	**All** ^**1**^	**Genotyped** ^**2**^
Year of birth^3^	2001	2004	2002	2005
Pedigree^4^	0.028 (0.022)	0.033 (0.017)	0.030 (0.027)	0.024 (0.023)
GRM_F^5^		0.134 (0.025)		0.144 (0.028)
Homozygosity^6^		0.653 (0.011)		0.715 (0.012)

All^1^: Cows with only pedigree inbreeding information; Genotyped^2^: the genotyped subset; Year of birth^3^: Mean of year of birth; Pedigree^4^: Mean of pedigree inbreeding coefficients; GRM_F^5^: the diagonal of the GRM – 1; Homozygosity^6^: proportion of the genome that is homozygous.

**Figure 3:**
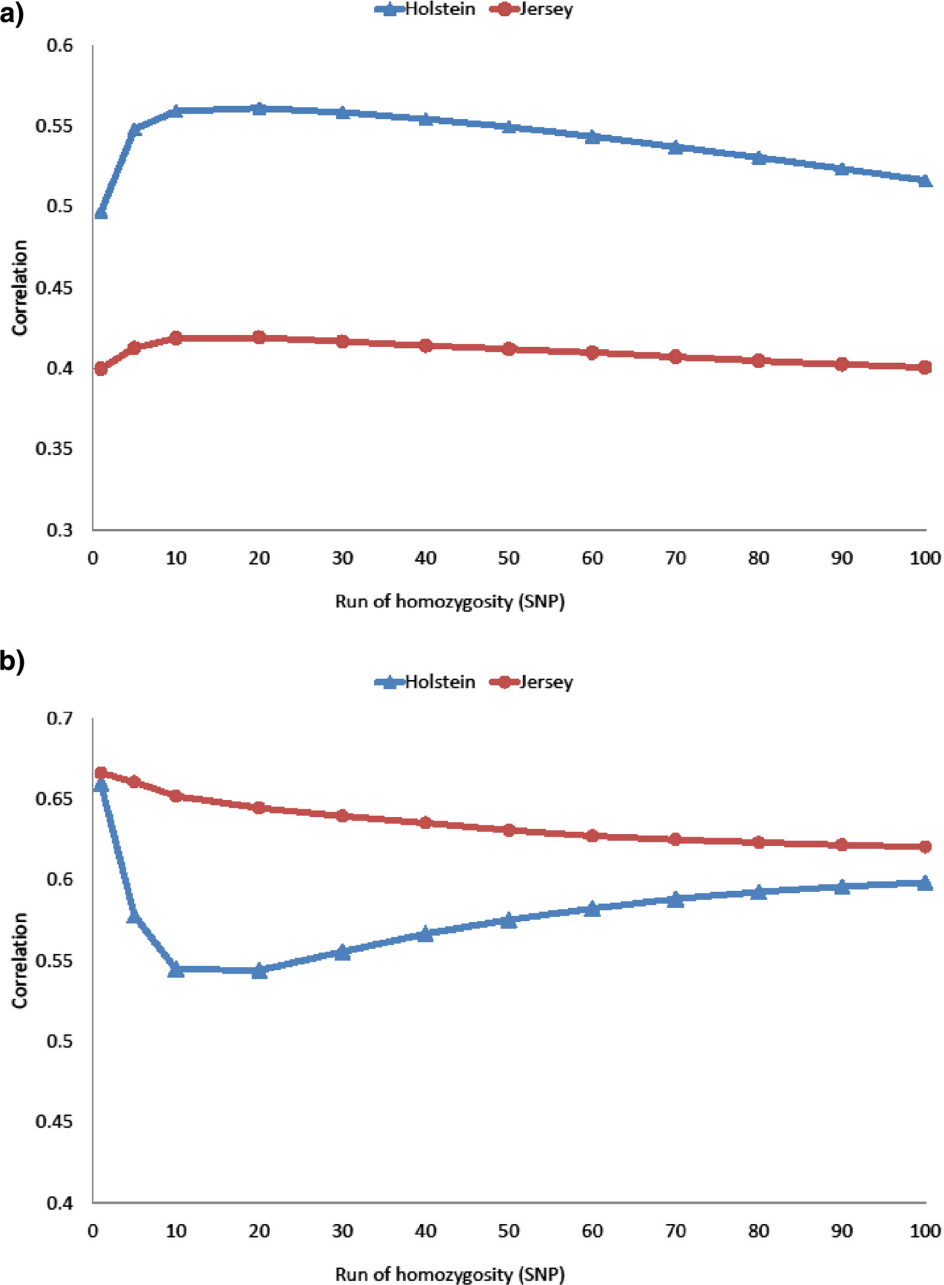
**Correlations between runs of homozygosity and pedigree and genomic inbreeding.** Runs of homozygosity refers to the proportion of an individual’s genome that exceeds the number of consecutive SNPs on the horizontal axis. **(a)** Correlations between runs of homozygosity and pedigree inbreeding. **(b)** Correlations between runs of homozygosity and genomic inbreeding, where genomic inbreeding is the diagonal of the genomic relationship matrix.

Correlations of pedigree-based inbreeding coefficients with each measure of genomic inbreeding (i.e., GRM_F, proportion homozygous, ROH_F) were lower than correlations among the genomic measures of inbreeding (Table 3). The correlation of the proportion of the genome that was homozygous with inbreeding coefficient GRM_F was equal to 0.72 for Holstein and 0.66 for Jersey animals. The correlation of ROH_F with the pedigree-based inbreeding coefficient was largest when the number of SNPs in the ROH_F was equal to 10 (Figure 3a) for both breeds. However, for the Holstein population, the correlation between GRM_F and ROH_F was greater for ROH equal to 1 (0.66), i.e., the proportion of SNPs that are homozygous, than for ROH equal to 10 (0.54) (Figure 3b). For the Jersey population, the correlations between ROH_F and GRM_F were more consistent and ranged from 0.66 for ROH equal to 1 to 0.62 for ROH equal to 10 (Figure 3b).

**Table 3:** **Correlations between pedigree and genomic inbreeding coefficients in Holstein and Jersey populations**

	**Pedigree**	**GRM_F** ^**1**^	**Homozygosity** ^**2**^	**ROH_F** ^**3**^
Pedigree	-	0.29^4^	0.45	0.53
GRM_F	0.26	-	0.72	0.65
Homozygosity	0.41	0.66	-	0.90
ROH_F	0.51	0.62	0.91	-

GRM_F^1^: the diagonal of the GRM – 1; Homozygosity^2^: the proportion of the genome that is homozygous (homozygosity); ROH_F^3^: runs of homozygosity of 50 SNPs or more; ^4^Correlations in the Holstein population are above the diagonal and correlations in the Jersey population are below the diagonal.

### Inbreeding depression

For Holstein cows, a 1% increase in pedigree-based inbreeding or in GRM_F was associated with a reduction in milk yield of 21 and 28 L/lactation, respectively (Table 4), which represents 0.3% to 0.4% of the phenotypic mean, respectively. For Jersey cows, a 1% increase in pedigree-based inbreeding or in GRM_F was associated with a reduction in milk yield of 12 and 27 L/lactation, respectively (Table 4), which represents 0.2% and 0.5% of the phenotypic mean, respectively. The effect of inbreeding on fertility was only significant (P <0.05) for the Holstein breed and when using pedigree-based inbreeding coefficients, where a 1% increase in inbreeding was associated with an extension of calving interval of +0.18 d, which represents 0.04% of the phenotypic mean (Table 4). Compared to the estimates of inbreeding depression based on the population that had only pedigree information, inbreeding depression effects were generally lower in the genotyped population. Furthermore, in the genotyped population, the effect of inbreeding depression was 2 to 3 times greater based on GRM_F compared to pedigree-based inbreeding for both breeds.

**Table 4:** **Effect on milk production traits and fertility in Holstein and Jersey populations of increasing inbreeding by 1%**

**Breed**	**Trait**	**Pedigree** ^**1**^		**Genotyped** ^**2**^			
		**Ped_F** ^**3**^		**Ped_F**		**GRM_F** ^**4**^	
		**b** ^**5**^ **(s.e.)**	**-log10 (P-value)**	**b (s.e.)**	**-log10 (P-value)**	**b (s.e.)**	**-log10 (P-value)**
Holstein	Milk (L)	-21.1 (1.5)	45.2	-11.3 (8.8)	0.7	-27.8 (5.7)	5.9
	Fat (kg)	-0.73 (0.06)	33.4	-0.67 (0.36)	1.2	-1.28 (0.23)	7.3
	Protein (kg)	-0.63 (0.05)	37.2	-0.33 (0.29)	0.6	-0.93 (0.19)	5.9
	Calving interval (d)	0.18 (0.05)	4.4	0.12 (0.26)	0.2	0.22 (0.17)	0.69
Jersey	Milk (L)	-12.0 (2.4)	6.4	-9.4 (7.1)	0.8	-27.2 (5.2)	6.8
	Fat (kg)	-0.62 (0.12)	7.0	-0.65 (0.36)	1.2	-1.58 (0.26)	8.6
	Protein (kg)	-0.45 (0.08)	6.7	-0.39 (0.26)	0.9	-1.09 (0.19)	7.7
	Calving interval (d)	0.14 (0.09)	0.8	-0.32 (0.28)	0.6	-0.08 (0.22)	0.2

Pedigree^1^: Population with pedigree information only; ^2^Genotyped^2^: Population with pedigree and genomic information; Ped_F^3^: pedigree inbreeding; GRM_F^4^: genomic inbreeding, defined as the diagonal of the GRM – 1; b^5^: effect of a 1% increase in inbreeding and associated standard errors (s.e.) for Holstein and Jersey populations for milk, fat and protein yields and calving interval as a measure of fertility.

For most other traits, the effects of increasing GRM_F were similar for the Holstein and Jersey populations (Table 4). However, as a percentage of the phenotypic mean, the average effects of GRM_F on milk, fat and protein yields were greater for the Jersey breed (0.57%) than for the Holstein breed (0.41%).

A 1% increase in homozygous SNPs resulted on average in a 1% and 1.5% reduction in milk, fat and protein yields for Holstein and Jersey cows, respectively (Table 5). The association with fertility was significant for the Holstein breed (P <0.05) and was equal to 0.93 d per 1% increase in homozygous SNPs (Table 5). Increasing GRM_F by 1% increases homozygosity by on average 0.33%, so the effect of a 1% increase in homozygosity is expected to be about 3 times the effect of a 1% increase in GRM_F, which is approximately what we observed.

**Table 5:** **Regression coefficients of milk production traits and fertility on homozygosity in Holstein and Jersey breeds**

**Trait**	**Holstein**		**Jersey**	
	**b** ^**1**^ **(s.e.)**	**-log10(P-value)**	**b** ^**1**^ **(s.e.)**	**-log10(P-value)**
Milk (L)	-63.0 (14.9)	4.6	-71.0 (15.1)	5.6
Fat (kg)	-3.0 (0.6)	6.4	-3.9 (0.7)	6.5
Protein (kg)	-2.0 (0.5)	4.3	-2.8 (0.56)	6.9
Calving interval (d)	0.93 (0.43)	1.5	0.30 (0.6)	0.21

b^1^: Regression coefficient and associated standard error (s.e.) estimated using homozygosity defined as the proportion of homozygous SNPs for milk, fat and protein yields and calving interval as a measure of fertility.

**Figure 4:**
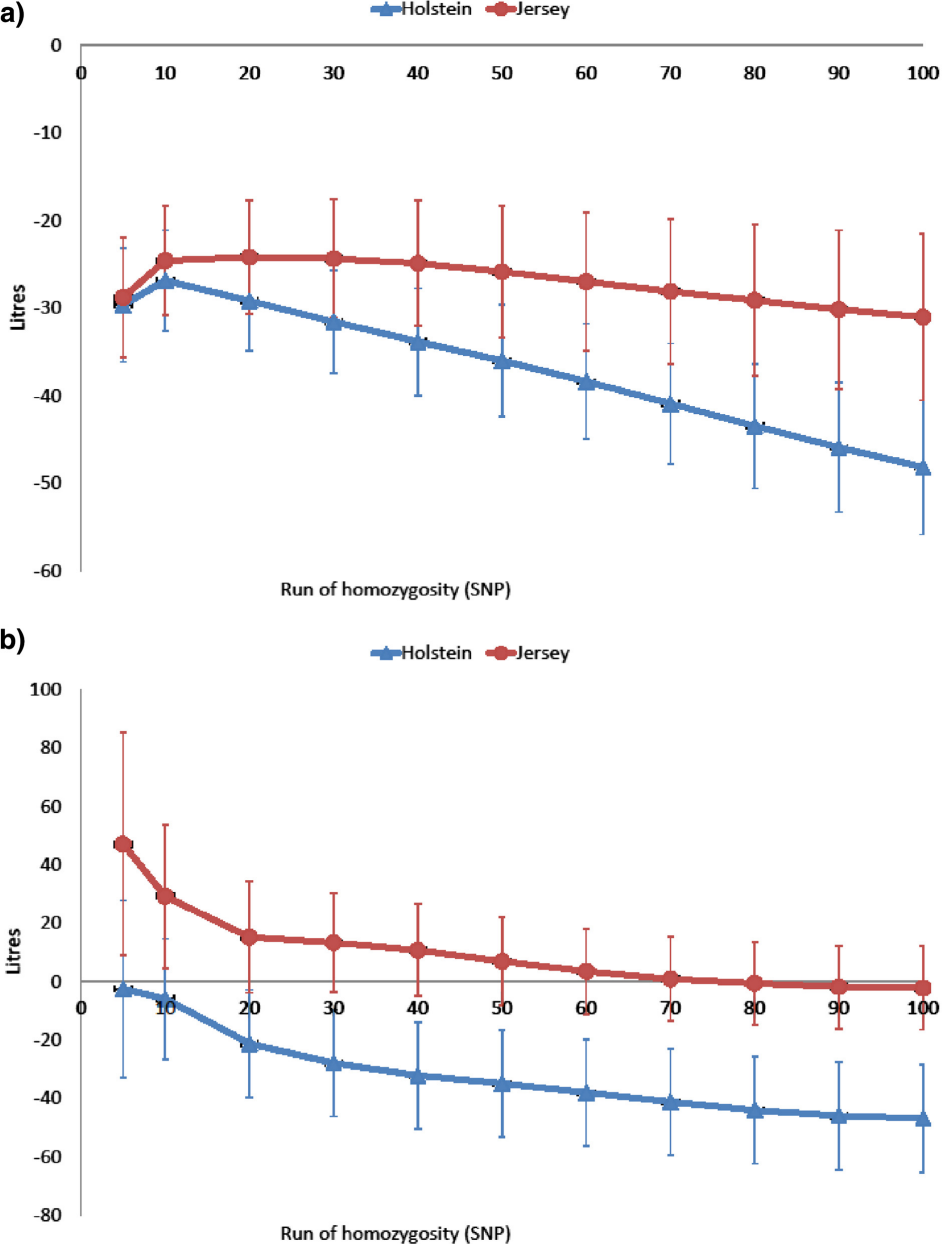
**Effect of runs of homozygosity on milk yield before (a) and after (b) correcting for overall homozygosity.**

Longer ROH, which indicates a more recent common ancestor, had a stronger unfavourable effect on milk yield than shorter ROH; a ROH of five SNPs led to a much smaller reduction in milk yield than a ROH of 100 SNPs (Figure 4a). However, general homozygosity may explain part of this relationship. To test this, the model was extended to include the overall level of homozygosity as an additional covariate. At the same level of overall homozygosity, shorter ROH (<60 SNPs) had no statistically significant association with milk yield in both breeds. However, longer ROH were associated with a reduction in milk yield that was independent of the proportion of the genome that was homozygous in the Holstein breed (Figure 4b).

The association between ROH_F (the proportion of the genome that consists of ROH) and fertility was not significant for either breed (results not shown). In fact, for the Holstein breed, the effect of ROH_F on fertility was almost entirely captured by the overall homozygosity.

### Genome-wide association studies

After correcting for the additive effect of SNPs, some regions of the genome contained stretches of ROH of 50 or more SNPs that were significantly associated with both milk yield and fertility for the Holstein and Jersey breeds. Significant ROH frequently formed clusters at overlapping positions on the genome, which may indicate that they were in linkage disequilibrium with the same QTL.

Negative associations of ROH with milk yield were found on chromosomes 11, 14, 16, 17, 20, 26, 28 for the Holstein breed and on chromosomes 8, 17, 20 and 24 for the Jersey breed (Table 6). Unfavourable associations of ROH with calving interval were found on chromosomes 2, 5, 8, 9, 15 and 24 for the Holstein breed and on chromosomes 24 and X for the Jersey breed.

**Table 6:** **Genomic regions associated with a reduction in fertility and milk yield identified using runs of homozygosity**

**Trait**	**Breed**	**Chr** ^**1**^	**Interval** ^**2**^ **(Mb)**	**Number of ROH P <0.001** ^**3**^	**Frequency of ROH** ^**4**^ **(%)**	**b (s.e)** ^**5**^	**-log10 (P-value)**
Fertility	Holsteins	2	1.34–1.69	7	6%	5.2 (1.5)	3.37
		2	13.0–13.5	2	5%	6.0 (1.8)	3.17
		5	37.6–37.7	3	5%	5.7 (1.6)	3.37
		8	88.0–88.5	5	6%	5.1 (1.5)	3.11
		9	6.9–9.2	26	3%	7.6 (2.1)	3.57
		15	24.7–28.6	6	4%	6.0 (1.8)	3.20
		24	60.4–60.5	3	4%	6.0 (1.8)	3.09
	Jerseys	X	60.4–62.1	14	4%	12.5 (3.6)	3.19
Milk	Holsteins						
		7	60.3–60.5	1	4%	-216 (65)	3.02
		11	99.9	2	3%	-245 (74)	3.03
		14	41.09525	1	3%	-255 (77)	3.00
		16	64.9–66.2	22	6%	-212 (58)	3.34
		17	70.7–70.8	2	4%	-260 (74)	3.34
		20	35.7–35.8	2	10%	-161 (48)	3.11
		26	32.3–33.9	12	4%	-239 (67)	3.29
		28	7.29–8.03	17	4%	-240 (66)	3.49
	Jerseys	8	89.7–95.5	27	7%	-254 (75)	3.16
		20	28.1–30.9	13	5%	-194 (55)	3.31
		24	19.3–19.5	3	5%	-234 (68)	3.21

^1^Chr = chromosome; ^2^Interval = interval between the start and end of the cluster of run of homozygosity (ROH) of 50 or more SNP from UMD build 3.1 measured in Mb; ^3^Number of ROH = the count of significant (P <0.001) ROH within the interval; ^4^frequency of ROH (%) = the mean frequency of ROH present in the interval; ^5^b = the regression coefficient of the phenotype (fertility which was calving interval; d or lactation milk yield L) on ROH and associated standard errors (s.e.).

For calving interval in Holstein cows, seven clusters of ROH were identified on different chromosomes. These clusters comprised a total of 63 ROH that were significant, of which 52 had an unfavourable effect. The false discovery rate (FDR) of the 63 ROH was estimated at 72.5% using the method of Bolormaa et al. [29]. The most probable real associations are likely to be the largest clusters; for example, there was a region on chromosome 9 in which 26 ROH had an unfavourable association with fertility in the Holstein population (Table 6). The size of this region was nearly 3 Mb (i.e. between 6.4 and 9.2 Mb) and included all the ROH (of 50 SNPs or more) present and led to an average increase in calving interval of 7.6 d (ranging from 6.7 to 8.4 d) in the Holstein population. For milk yield in the Holstein population, 117 ROH were significant (P <0.001), which corresponds to a FDR of 39%. Of these 117 ROH, 65 had an unfavourable association with milk yield and formed eight clusters. A group of 22 significant ROH (at P <0.001) was found on chromosome 16, which spanned a region between 64.9 and 66.2 Mb that contained 18 candidate genes (Table 6). On average, these ROH were associated with a reduction in milk yield of ~228 L/lactation. A region on chromosome 2 between 12.8 and 14.3 Mb that contained 24 ROH showed a significant (P <0.01) and positive (i.e. favourable) association with milk yield and an unfavourable association with calving interval. Although a more stringent significance level was necessary to detect this relationship, it is possible that there are many other regions of the genome with such opposing effects, since correlations between fertility and milk yield are generally unfavourable.

Since the Jersey population was smaller, there was less power to detect ROH that had an association with either milk yield or fertility. For this breed, only 44 and 17 ROH were significantly associated with milk yield and fertility, respectively, with FDR of 100% or more indicating that all these associations were probably identified by chance. However, 14 ROH on chromosome X had an effect on fertility in this breed (Table 6), with an FDR of only 6%, which suggests that this region is associated with inbreeding depression. Three regions on chromosomes 8, 20 and 24 were associated with milk yield in the Jersey breed, although none of these overlapped with those found for the Holstein breed. Detecting identical ROH with associations in two breeds is useful for validation, since it is unlikely that the same association is found in two breeds by chance. In our data, such cases were rare and only occurred when the significance threshold for the Jersey breed was relaxed to P <0.01, which is justified by its smaller population size. Of particular interest was a region found on chromosome 24 at 60 Mb that had an unfavourable association with fertility in the Holstein dataset (Table 6), which was validated in the Jersey dataset at P <0.01.

## Discussion

### Inbreeding coefficients

Correlations of 0.26 and 0.29 were found between inbreeding coefficients based on pedigree and GRM_F for the Jersey and Holstein datasets, respectively (Table 3). These values are lower than those observed for USA Holstein bulls (which ranged from 0.5 to 0.56; [30]) and for Australian Holstein bulls (which ranged from 0.67 to 0.87 for bulls with two to eight generations of recorded ancestry; [31]). There are three possible reasons why the correlations found in our study are lower than those reported in other studies: (1) individuals from sub-populations for which allele frequencies diverge from those of the entire population are estimated to have high inbreeding based on GRM_F; (2) pedigree completeness and (3) a possible bias introduced by the selection of the cows that were genotyped, since cows that had multiple lactation records were preferentially selected, which may have purged strongly deleterious effects out of this older population. In fact, the genotyped populations were on average 1.2 lactations older for the Holstein dataset and 0.6 lactations older for the Jersey dataset. So, purging may explain why pedigree inbreeding depression (Table 4) was on average 59% and 12.8% lower in the genotyped Holstein and Jersey populations, respectively, compared with the population that had only pedigree information. This comparison suggests that the effect of purging in the genotyped population could be greater than the value of 12.6% predicted by Gulisija and Crow [32].

Correlations of the proportion of homozygous SNPs with pedigree-based inbreeding coefficients were stronger than the equivalent correlations using GRM_F for both Holstein and Jersey populations. The lower correlations obtained with GRM_F are probably due to the effect of allele frequencies in the sub-population on the diagonal elements of the GRM, which results in artificially elevated “inbreeding coefficients” in minority sub-populations. To overcome this problem, several authors [14,30,33] have proposed calculating the GRM with allele frequencies fixed at 0.5. In fact, the resulting GRM is the same as the proportion of homozygous SNPs, since the correlation between these was equal to 0.99 [14]. Therefore, using either the proportion of homozygous SNPs or the equivalent measure of the diagonal of the GRM calculated with an allele frequency of 0.5 is preferred for the estimation of a base population.

In accordance with other studies, the correlations between pedigree and genomic measures of inbreeding in cows were lower than the equivalent estimates in bulls [30,31], which implies that pedigree recording was worse for females than for males. This is expected for commercial dairy populations and is exacerbated in countries like Australia where calving occurs in unsupervised outside conditions and mismothering of calves is a problem. Furthermore, partial or incomplete pedigrees also reduce pedigree inbreeding estimates [21]. Pedigree completeness was lower for the Jersey dataset than for the Holstein dataset, which probably explains why the correlation between proportion of homozygous SNPs and pedigree-based inbreeding coefficients was weaker for the Jersey than for the Holstein population. This is consistent with the fact that pedigree inbreeding tends to be lower for genotyped Jersey cows than for all Jersey and Holstein animals (Figure 1). However, inbreeding for all measures derived from genomic data was higher for the Jersey than the Holstein population. Furthermore, Jersey cows had on average a higher proportion of homozygous SNPs than Holstein cows (0.72 and 0.66, respectively) which is probably because (1) Jerseys were more inbred or (2) many of the SNPs on the genotyping panel were discovered using Holstein animals [25].

The correlation between ROH and pedigree-based inbreeding was strongest for ROH that consisted of 10 SNPs (0.58 Mb) (Figure 4a) and stronger than the correlation of any other genomic measure with pedigree-based inbreeding (Table 3). In the absence of pedigree data, the proportion of the genome that comprises ROH of 10 SNPs or more can be used to infer recent common ancestors [16,34], which makes ROH an attractive tool to monitor and evaluate inbreeding depression.

### Inbreeding depression

A 1% increase in inbreeding in the population with pedigree information only, or in the population with GRM_F resulted in a reduction in milk production traits of around 0.3% of the phenotypic mean and a deterioration in fertility for both the Holstein (Table 4) and the Jersey breeds. Comparable results have previously been published using pedigree-based inbreeding; a 1% increase in inbreeding has been estimated to be associated with a reduction in milk yield of around 20 to 30 L/lactation [35-37,6,7] and a lengthening of calving interval of up to 0.7 d [6,38]. The small effect of inbreeding on calving interval may be due to the tendency of Australian farmers to cull cows that are expected to calve late, causing a downward bias in the effect on calving interval.

There have been relatively few studies on inbreeding depression using genomic measures of inbreeding. Recently, Bjelland et al. [14] estimated inbreeding depression using a measure similar to GRM_F and found a reduction in milk yield of 47 L/lactation and an increase of 1.06 d for days open (which is a trait very similar to calving interval) per % increase in inbreeding. The results presented in our study and those of Bjelland et al. [14] show that genomic estimates of inbreeding can be used instead of pedigree estimates to calculate the effects of inbreeding on performance and fitness traits. Under ideal conditions, the effect of pedigree inbreeding and GRM_F would be the same. However, errors in pedigree records are expected to reduce pedigree-based estimates of inbreeding depression. Estimates based on GRM_F should have a lower sampling error than those based on pedigree because it is not affected by incomplete pedigrees and it uses observed rather than expected IBD. However, this benefit had less effect on standard errors obtained in this study than the large difference in sample size used for the pedigree and genomic analyses. The magnitude of the difference in inbreeding depression estimated based on GRM_F and based on pedigree information for the genotyped cows implies that estimates of inbreeding depression on traits of economic importance may in fact be underestimated using pedigree information.

The proportion of homozygous SNPs was associated with inbreeding depression in all traits analysed, with the exception of fertility in the Jersey breed, which could be due to a lack of statistical power and bias in calving interval caused by culling. However, the association between homozygosity and milk production was not stronger than that between GRM_F and milk production (Tables 4 and 5). The allele frequencies of SNPs contain information on the probability that chromosome segments are IBD because segments that share a rare allele are more likely to be IBD than segments that share a common allele. Thus, including the base population in the calculation of inbreeding (i.e. GRM_F) can be advantageous. However, the correlation between pedigree-based inbreeding and SNP homozygosity was higher and as previously discussed, sub-populations that have allele frequencies that differ markedly from those of the base population appear to be more inbred than they actually are. Therefore, GRM_F has both advantages and disadvantages compared to SNP homozygosity as a measure of inbreeding. These advantages and disadvantages appear to approximately compensate each other, so while homozygosity may be a better indicator of pedigree-based inbreeding, it appears that GRM_F and homozygosity are equally good indicators of inbreeding depression.

Long ROH (>60 SNP or 3.5 Mb) were associated with a decrease in milk yield after correcting for average homozygosity, but short ROH were not. This could be due to selection eliminating deleterious mutations before inbreeding occurs if the inbreeding is due to an ancient common ancestor. Inbreeding arising from recent common ancestors has also been found to be associated with reproductive performance and body weight in mice [17] and with a large decrease in height in humans [30].

To account for recent and older inbreeding simultaneously in breeding programs that aim at reducing inbreeding, longer ROH (>50 SNPs or 3 Mb) should be explicitly considered in addition to other measures of genomic inbreeding. In this study, the Illumina BovineSNP50 BeadChip (Illumina, San Diego, CA; [25]) was used because of its popularity to estimate genomic breeding values [39]. However, while this chip may be suitable for genomic evaluations, it has been reported to overestimate the number of ROH present compared to the Illumina HD panel [40]. However, both SNP panels are equally effective at detecting segments that are longer than 4 Mb, which corresponds to approximately 70 SNPs [40]. Thus, a negative aspect of our study could be that short ROH are not necessarily IBD. However, relying on longer ROH has the disadvantage that they have a lower frequency (Figure 2) and thus fail to identify small IBD regions. An area for future research could be to compare inbreeding effects of ROH obtained with high-density chips, or even whole-genome sequences with those obtained with lower density SNP panels.

### Genome-wide association study

For the GWAS analysis, a ROH length of at least 50 SNPs (~3 Mb) was chosen because, in this case, the ROH frequency was large enough to detect statistically significant differences and the ROH length was sufficient to detect recent inbreeding. Other studies [19] have reported that using the Illumina BovineSNP50 BeadChip with ROH length set at 50 SNPs allowed the detection of fragments of more variable size (especially those less than 5 Mb) when compared to ROH of 50 SNPs. After correcting for the additive effect of the SNP at a given position, using a GWAS analysis, we still detected an effect of ROH on either calving interval or milk yield of up to 12.5 d and 260 L, respectively. This implies that there is considerable value in selecting for heterozygosity in regions where homozygosity has an unfavourable effect on valuable traits, such as fertility and milk yield. However, although our analysis identified several regions of interest, the associated FDR were high, which means that further validation is required.

The regions that were identified here were not close to the recessive lethal haplotypes reported by VanRaden et al. [41] and Fritz et al. [42], for which no homozygotes were observed. This is not surprising, since the homozygous regions detected in our study were present in the population and therefore, were unlikely to have a lethal effect.

However, the ROH that were identified as significant could be associated with genes that, if impaired, can lead to loss-of-function. In addition, when the presence of a ROH has a positive effect on a trait under selection, such as milk yield or fertility, it can be indicative of selection signatures [19].

For the Holstein population, a region on chromosome 16 contained 22 ROH that had a negative effect on milk volume. This region included 18 genes (see Additional file 1). The most promising candidate gene in this region is *glutamate-ammonia ligase* (*GLUL*), which is involved in alanine, aspartate, and glutamate metabolism, arginine and proline metabolism and nitrogen metabolism. This gene has previously been reported to control fat to protein ratio [43]. Another region on chromosome 28 that affected milk yield contained eight genes. Bouwman et al [44] reported the identification of SNPs associated with milk fatty acid composition in this region.

On chromosome 20, two ROH were detected that, when homozygous, had a negative effect on milk yield in both the Holstein and Jersey breeds. Selection signatures close to this region have been identified in the genomes of US and German Holsteins [19,45] and the *growth hormone receptor* (*GHR*) gene has been proposed as the likely candidate gene [45], although some publications suggest other possible candidates, such as *prolactin receptor* (*PRLR*) [46,47]. Neither *PRLR* nor *GHR* aligned with the genes identified in our scan. One hypothesis is that intense selection for *GHR* or *PRLR* has made this genomic region globally more homozygous and while homozygosity of specific alleles may be beneficial, overall homozygosity is detrimental to production because of unfavourable alleles that are in linkage disequilibrium with the favourable alleles of *GHR*/*PRLR*.

For the Jersey breed, there was a cluster of 14 ROH on the X chromosome, located between 60.4 and 62.1 Mb (Table 6), that were significantly (P <0.001) associated with fertility. A candidate gene in this region (*insulin receptor substrate 4*; *IRS4*) has been reported to be associated with mild defects in reproduction and growth in mice [48]. Insulin receptor substrates mediate the actions of insulin and control energy balance and glucose homeostasis [49].

One of the strengths of this study was that two breeds, Holstein and Jersey, were used. Thus, any effects that were detected in one breed and validated in the other are more likely to be true rather than false discoveries. Unfortunately, there was only one region (on chromosome 24 at around 60 Mb), with an unfavourable association with fertility, that was validated in both breeds. No candidate genes were identified in this region (see Additional file 1), although the ROH could have been in linkage disequilibrium with QTL that were located further away. Our understanding of genomic inbreeding will improve as datasets increase in size and the ability to find causative mutations will also increase as a result of high-density genotyping and sequencing.

One potential use of non-additive effects is to predict the future performance of a cow or of the potential heifer resulting from a planned mating. This prediction would use both the additive and dominance effects and should reduce the production of calves that are homozygous for deleterious mutations. In the past, customised mating programs have focused on genome-wide inbreeding [31,50] but, with the availability of genomic data, information on the genomic locations for which deleterious recessive alleles segregate, could be used.

## Conclusions

Genomic measures of inbreeding can be used instead of pedigree-based inbreeding to estimate the effects of inbreeding depression and give similar estimates. Milk, fat and protein yields and fertility were unfavourably impacted by increasing genomic and pedigree measures of inbreeding for the Holstein and Jersey breeds of dairy cattle. Although inbreeding was higher for the Jersey than the Holstein breed, the effect of increasing inbreeding on the traits studied was similar for both breeds. In situations where sub-populations are suspected, it may be preferable to consider a measure of inbreeding that does not depend on allele frequencies, such as the proportion of homozygous SNPs. However, in our data, homozygosity did not have a larger effect on milk production and fertility than the diagonal element of the GRM. ROH can provide additional benefits, since longer ROH (>3 Mb) capture recent inbreeding, which had an effect independent of overall homozygosity on milk production. Using a GWAS with ROH of 50 or more SNPs, genomic regions were detected that had an effect of up to 12.5 d and 260 L for calving interval and milk yield, respectively, when completely homozygous. Breeding programs could exploit whole-genome data, as well as these site-specific regions.

## Additional file

Additional file 1:
**List of genes and their positions (UMD 3.1) located within the runs of homozygosity that were found to be associated with milk yield and fertility in the Holstein and Jersey breeds.** Ensembl gene identifications, chromosome number, start and end position of the gene on the chromosome in base pairs, strand, gene name and associated description for genes that were located within runs of homozygosity found to be associated with milk yield and fertility in the Holstein and Jersey breeds accessed from Ensembl BioMart MartView in June 2014 (http://asia.ensembl.org/biomart/martview/).
